# A Nanoplasmonic Strategy for Precision *in-situ* Measurements of Tip-enhanced Raman and Fluorescence Spectroscopy

**DOI:** 10.1038/srep19558

**Published:** 2016-01-19

**Authors:** Lingyan Meng, Mengtao Sun, Jianing Chen, Zhilin Yang

**Affiliations:** 1 Department of Physics, Xiamen University, Xiamen 361005, China; 2 Beijing National Laboratory for Condensed Matter Physics, Institute of Physics, Chinese Academy of Sciences, Beijing, 100190, China; 3 Collaborative Innovation Center of Quantum Matter, Beijing 100871, China

## Abstract

We theoretically investigate an optimized tip-film system that supports *in-situ* measurement of tip-enhanced Raman spectroscopy (TERS) and tip-enhanced fluorescence (TEF) of dye molecules. A scanning tunneling microscope (STM) is proposed to precisely control the tip-film distance, and thus *in-situ* measurement of TERS and TEF can be realized utilizing the specific surface plasmon resonance (SPR) properties of the tip-film system. Our calculations show that the optimized tip-film distance of 2 nm suggests a possibility of efficient acquisition of TERS and TEF *in-situ*. The calculated spatial resolution of TERS and spectral resolution of TEF can be down to 6.5 nm and 10 nm, respectively. Our theoretical results may find promising application in developing multiple functional nano-spectroscopy through which Raman and fluorescence can be measured *in-situ* at the nanoscale level.

Localized surface plasmon resonance (LSPR) gives rise to strong electromagnetic (EM) field enhancement in the vicinity of metallic nanostructures. The localized EM field strongly enhances the light-matter interaction in the near-field, and results in surface-enhanced phenomenons such as surface-enhanced Raman scattering (SERS)^1^,^2^,^3^ and surface-enhanced fluorescence (SEF)^4^,^5^. SERS is an ultrasensitive spectroscopic analytical technique for detecting Raman signals of molecules with intrinsic small Raman cross section, and the maximum of SERS enhancement can be up to 12–14 orders^6^. SERS usually accompanies by surface-enhanced fluorescence (SEF) phenomena, which is from the coupling between the absorption band of fluorophore and LSPR of the metallic nanostructures. However, the theoretical maximum of SEF enhancement factor is only 1–3 orders^7^,^8^. This low enhancement factor is the consequence of competition between quenching and enhancing of fluorescence^9^, which is particularly sensitive to the fluorophore-metal distance^10^,^11^, Nonradiative energy transfer causes the quenching of fluorescence and the enhanced EM field by SPR provides the enhancing of fluorescence. SERS occurs when the molecules are in the vicinity of metal, however fluorescence gets quenched in the same condition. Therefore most experiments deal with surface-enhanced Raman scattering (SERS) or surface-enhanced fluorescence (SEF) independently. SERS provides vibrational or rotational energy state of a molecule, and fluorescence provides information of energy level and excitation spectra of molecule^12^. The combination of SERS and SEF will contribute to fill the gaps that these two spectroscopic techniques cannot cover independently. It is tempting to have a technique that allows *in-situ* SERS and SEF measurements. However, few works realized the precision *in-situ* measurements of Raman and fluorescence spectra^13^,^14^,^15^,^16^. The difficulties are controlling the distance between the fluorophore and metal nanostructures and the stronger fluorescence background than Raman signal.

Compared with the traditional SERS and SEF, tip-enhanced Raman spectroscopy (TERS)^17^,^18^,^19^ and tip-enhanced fluorescence (TEF)^20^,^21^,^22^ have the advantages of high spatial and spectral resolution, and the ability to manipulate a probe for varying parameters of experiment. Due to the strong coupling property of the tip-film system, the enhanced EM can well suppress the quenching while the molecules are in the vicinity of tip, and thus results in a considerable enhancement of TEF. In addition, the spatial resolution of TERS can be down to sub-nanometer resolution^17^, and the spectra resolution of TEF can be down to ~10 nm^23^. The combination of TEF and TERS is highly desirable for improving the sensitivity and accuracy in the detection technology.

From the instrument design point of view, we will optimize the design of TERS and TEF configurations to realize the combination of TERS and TEF techniques which is of very importance to develop multiple functional nano-spectroscopy at the nanoscale level. If it is feasible, the *in-situ* precise measurements of TERS and TEF can come true. The three-dimensional finite-difference time-domain (3D-FDTD) method was used to study the SPR properties of a conical silver tip on a silver film where Rhodamine 6G (R6G) was selected as the target molecules, under the side illumination mode. Our calculations show that *in-situ* measurements of TERS and TEF can be achieved by proper modeling.

## Results

**Figure 1:**
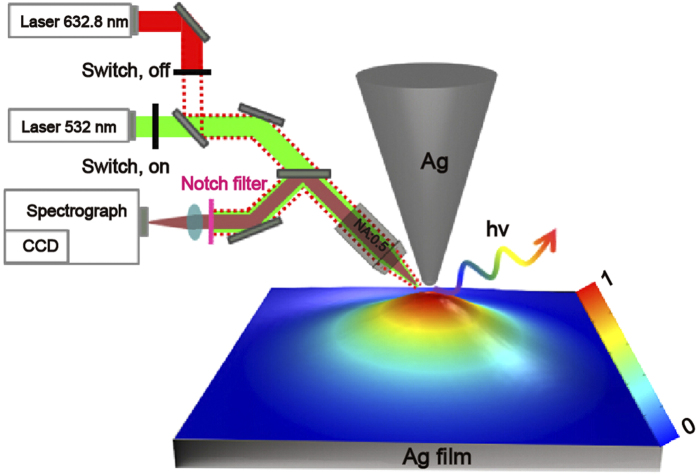
The schematic of *in-situ* measurements of TERS and TEF. The color map shows the schematic of the electric field intensity distribution of the plane between the tip and film.

In this work, the scanning tunneling microscope (STM) based TERS and TEF configurations were chosen as our research systems in which the tip-film distance can be precisely controlled. It should be noticed that other scanning probe microscopy (SPM) methods, such as atomic force microscopy (AFM), can also offer precise nano-positioning control as well as strong electromagnetic field enhancement^24^. The simulation configuration for TERS and TEF consists of a silver conical tip over an Ag film, as depicted in Fig. 1. The color map shows the schematic of the electric field intensity distribution of the plane between the tip and film, The final radius and the full cone angle of the tip, and the tip-film separation is 25 nm, 20° and d, respectively. Two p-polarized plane waves are focused from the side at an angle of 60° to the nanogap formed by the tip and film, and the electric field amplitude of the incident beams are set to be 1.0 V/m.

**Figure 2:**
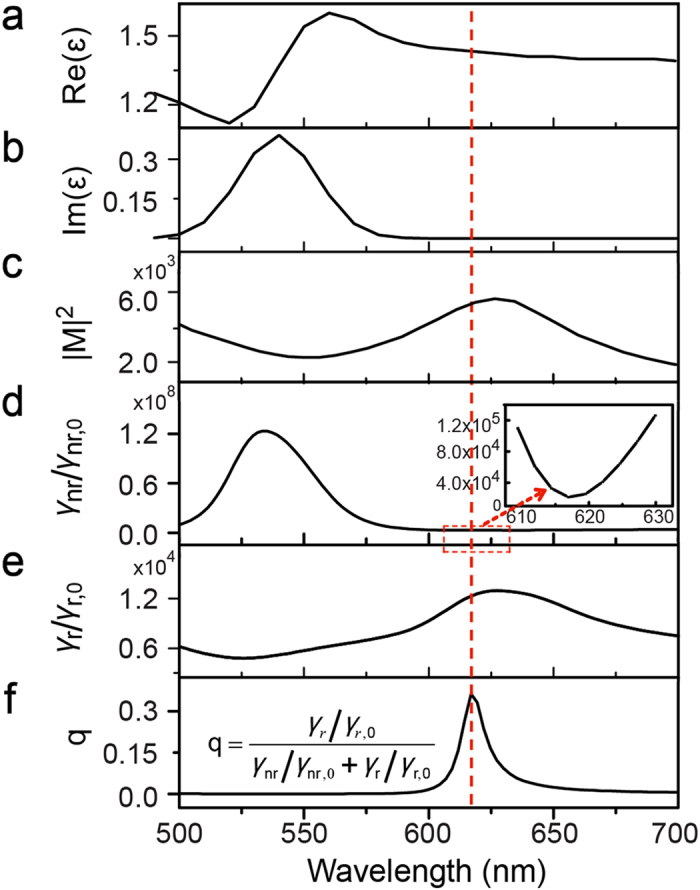
(**a**) The real (Re(ε)) part and (**b**) the image (Im(ε)) part of relative dielectric function (ε) of R6G adsorbed on Ag film. (**c**) The wavelength dependent electric field enhancement (|M|^2^) in the nanogap between the Ag tip and Ag film. (**d**) FDTD calculated nonradiative decay rate enhancement (γ_nr_/γ_nr,0_), and (**e**) radiative decay rate enhancement (γ_r_/γ_r,0_), and (**f**) quantum yield (**q**) of R6G in the coupled tip-film system, plotted as a function of wavelength. Inset: enlarged image of the region between 610 nm and 630 nm in (**d**).

Figure 2a,b show the real (Re (ε)) and imaginary (Im(ε)) parts of relative dielectric function (ε) of R6G adsorbed on Ag film^25^. The large Im(ε) around 540 nm demonstrates a strong molecular absorption. The 532 nm laser is chose to be the light source for molecule fluorescence in the coupled tip-film system, which can provide enough photon energy for exciting R6G from ground state to excitation state. Although the excitation energy is far from the LSPR peak in Fig. 2c, there is still strong EM enhancement around 532 nm with |M|^2^ = 2.6 × 10^3^, which can strongly enhance molecule absorption. We calculated the electric field enhancement |M|^2^ (defined as |E_loc_/E_in_|^2^, where E_loc_ and E_in_ are the local and the incident electric field, respectively) at the height of 0.5 nm above the surface corresponding to the height of R6G molecule film. As shown in Fig. 2c, the strong LSPR around 630 nm leads to a maximum TERS enhancement, which is proportional to |M|^4^. The calculated nonradiative decay rate enhancement (γ_nr_/γ_nr,0_) (see Fig. 2d) and radiative decay rate enhancement (γ_r_/γ_r,0_) (see Fig. 2e), are normalized respectively by that of target molecules in free space, which shows that nonradiative decay plays a dominant role in the shorter wavelength range while the radiative decay dominate in the longer wavelength range^8^. Furthermore, the strong peaks for γ_nr_/γ_nr, 0_ around 540 nm and γ_r_/γ_r, 0_ around 630 nm indicate that the nonradiative coupling between the excited molecule and the tip-film system leads to Förster energy transfer from the excited molecule to the tip-film system around 540 nm^26^,^27^, and then the transferred energy can be subsequently radiated around 630 nm by the tip-film system. Comparing Fig. 2c,e, the excitation wavelength of maximum γ_r_/γ_r, 0_ around 630 nm matches the maximum of |M|^2^, which reveals that the radiative decay is strongly enhanced by LSPR in the coupled tip-film system.

To obtain the quantum yield (q), we have studied the radiative and nonradiative decay rate enhancement of molecules in the tip-film system. The quantum yield (q) can be estimated with^7^,


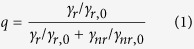


Figure 2f demonstrates that there is a large quantum yield (*q*) of R6G in the coupled tip-film system around 620 nm. We can conclude that both the radiative decay rate enhancement (γ_r_/γ_r,0_) and quantum yield (q) can be improved by the LSPR in the coupled tip-film system, which reveals a strong TEF enhancement.

In a tip-film configuration, the electromagnetic field enhancement and the spatial resolution were governed by many variables such as the tip and film material, the tip size and shape, the tip-film distance, and the incidence angle and polarization of the excitation light^28^,^29^. For example, the optimal incidence angle for a maximum electric field enhancement is in the range of 40–60° for a gold conical tip above an gold film. The spatial resolution is mainly dependent on the tip size and the tip-film distance. A decrease of the tip radius can improve dramatically the spatial resolution. Changing the metallic film into a dielectric film will lower the electric field enhancement and the spatial resolution. Here we optimize the design of TERS and TEF configurations to realize the combination of TERS and TEF techniques from the point of view of tip-film distance that is the dominating effect providing tremendous electromagnetic field enhancement and largely improving the spatial resolution.

**Figure 3:**
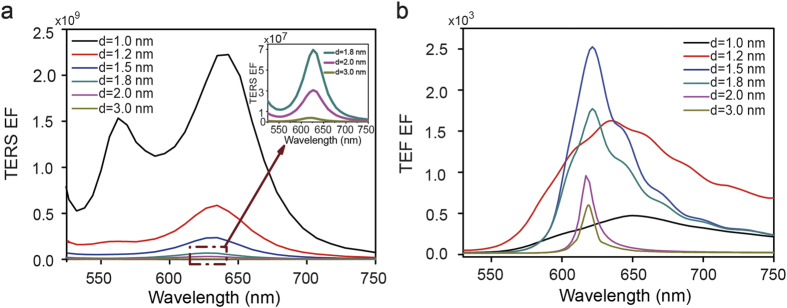
(**a**) Dependence of TERS enhancement factor (EF) and (**b**) TEF enhancement factor (EF) on wavelength with varying tip-film distance.

The strong near-field coupling effect between the tip and the film provides a huge electric field enhancement^29^, and thus enhances the TERS and TEF signals of target molecules. The tip-film coupling is extremely sensitive to the distance between the tip and film. Optimizing the distance is the key to measure TERS and TEF spectra *in-situ*. Figure 3a shows the calculated TERS enhancement factor (EF) (|M|^4^ = |E_loc_/E_in_|^4^) as a function of incident wavelength and tip-film distance. For a tip-film distance of 1 nm, we observe two distinct resonance behaviors which are attributed to the vertical (long wavelength) and the horizontal (short wavelength) dipole resonance respectively. As increasing the tip-film distance, the two resonances gradually shift to the blue and the TERS EF significantly decrease. The variation tendency of TERS EF with the tip-film distance is good agreement with published experimental results^30^,^31^. The horizontal dipole resonance vanishes when the tip-film distance increases to 1.5 nm because of the weak electromagnetic coupling between the tip and film in the horizontal orientation. The maximum TERS EF is 2.2 × 10^9^ for the tip-film distance of 1 nm with excitation wavelength of 642 nm. It should be noticed that in all our calculations, the tip-film distance is at least 1 nm in which the electron potential between the tip and the film is characterized by a large potential barrier that prevents electrons from transferring between the tip and the film. In this case, the electron tunneling effect is very weak^32^,^33^. In all our calculations, the electron tunneling effect was not considered.

We also observe decreasing the tip-film distance can provide a higher off-resonance enhancement factor than the on-resonance one for larger tip- film distance. In a TERS system, the strongly near-field coupling between the tip and the film has dominating contribution to the TERS enhancement. As decreasing the tip-film distance, the increased near-field coupling improves the enhancement factor in the whole spectrum region including the on-resonance and off-resonance region. Furthermore, the enhancement factor exponentially decreases with increase of tip-film distance. So, the TERS enhancement factor is always much larger for shorter tip-film distance, even for the off-resonance region.

According to Fig. 2c, the LSPR band is around 632.8 nm, but the photon energy is not sufficient to excite R6G molecules. We thus use 532 nm as the excitation wavelength, which provides high photon energy as well as strong EM enhancement. Figure 3b shows the calculated TEF EF, which is defined as |M|^2^(λ_ex_)q(λ_em_) where λ_ex_ and λ_em_ are the excitation and emission wavelengths, as a function of incident wavelength and tip-film distance. Around the LSPR band in Fig. 2c, the quantum yield is greatly enhanced. Considering the comprehensive effect of electric field enhancement and quantum yield, the fluorescence enhancement is still large at other excitation wavelengths (around 620 nm), even there is no strong electronic adsorption. In all cases we observe resonance behaviors around 620 nm. As increasing the tip-film distance from 1 nm to 1.5 nm, the TEF EF increases rapidly, and then the maximum TEF enhancement is obtained at a tip-film distance of 1.5 nm showing TEF EF of 2.5 × 10^3^ at excitation wavelength around 620 nm. If we are further to increase the tip-film distance, the TEF EF decreases rapidly. The simulated results is similar to a previous experimental results where a silicon tip was used for the fluorescence enhancement and the CdSe-ZnS core-shell quantum dots was use as the samples^20^. The differences between these two kinds of tips lie in the optimal distance. The optimal tip-film distance in the Ag tip-Ag film configuration is 1.5 nm while the tip-sample distance in the silicon tip-glass configuration is 0 nm. That’s because silver has larger quenching effect for the fluorescence molecules than silicon, and thus cause decrease of TEF EF when the tip-film distance is less than 1.5 nm.

## Discussion

**Figure 4:**
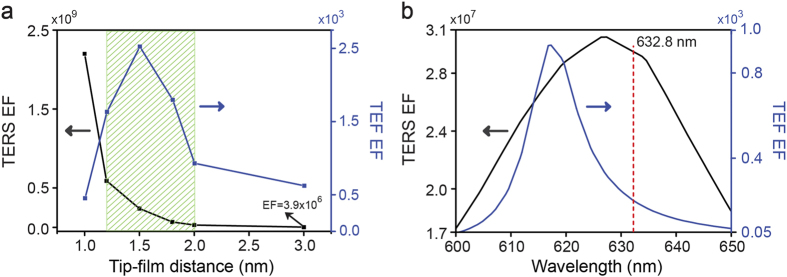
(**a**) Dependence of TERS and TEF enhancement factor on tip-film distance. (**b**) Dependence of TERS and TEF enhancement factor on wavelength at tip-film distances of d = 2 nm. The shadow in Fig. 4(a) stands for the suitable region for *in-situ* measurement of TERS and TEF. The red dotted line is 632.8 nm laser.

From the above results, the TERS and TEF enhancements are highly sensitive to the tip-film distance. To find the optimal distance for *in-situ* measurement of TERS and TEF, two critical factors should be considered as the criterion. The first factor is the enhancement factor of TERS and TEF. In Fig. 4a, we compared the dependence of TERS EF excited at 632.8 nm and TEF EF around 620 nm on the tip-film distance. We observe the TERS enhancement decays in an exponential with increasing tip-film distance, and the TEF enhancement increase with increase of the tip-film distance from 1 nm to 1.5 nm and then decrease as further increasing the tip-film distance. By comparing the TERS and TEF enhancement, we find the best tip-film distance for *in-situ* measurement of TERS and TEF should be in between 1.2 nm and 2.0 nm. However, when we consider the influence of fluorescence background in Raman spectroscopy in a real experiment, the full width at half maximum (FWHM) of TEF spectra, which is the second critical factor, need to be narrow enough to avoid overlap between the surface plasmon resonance (SPR) bands of TERS and TEF. As a result, the Raman detected region can be far from the fluorescence detected region, and thus the interference fluorescence background to the Raman signal can be well suppressed. Considering above factors, our theoretical calculations demonstrated that the best tip-film distance for *in-situ* precision measurement of TERS and TEF is 2 nm, as shown in Fig. 4b. We observe a maximum TERS enhancement around 3.0 × 10^7^ with excitation wavelength of 632.8 nm. The maximum TEF EF is enhanced to 940 around 620 nm where the Raman disturbance is avoided. It should be noted that the Raman excitation region is actually partially overlaped with the fluorescence peak region. But the fluorescence background is weak enough to avoid the fluorescence disturbance.

**Figure 5:**
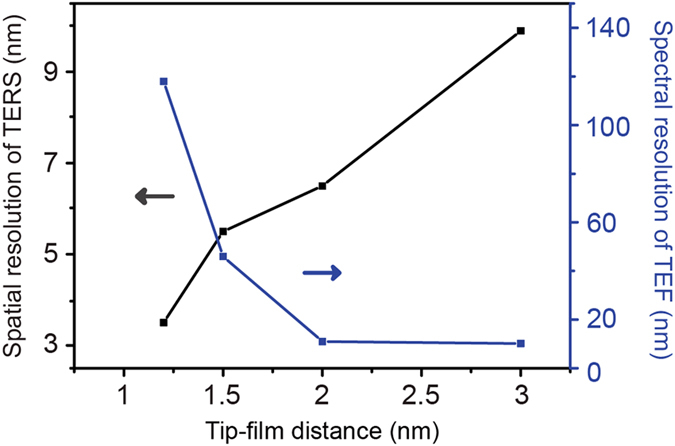
Dependence of spatial resolution of TERS and spectral resolution of TEF on tip-film distance.

Improving of spatial resolution is an important issue for achieving highly resolved optical images of target molecules. Although a sub-nanometer resolution has been demonstrated under ultrahigh vacuum and low temperature^17^, it is still a challenge to further improve the spatial resolution. The spatial resolution is quantified as the full width at half-maximum (FWHM) of the TERS enhancement along a horizontal line 0.5 nm on top of a silver film below the apex of a silver tip. Figure 5 shows the dependence of spatial resolution of TERS on the tip-film distance. We observe the spatial resolution increases as decreasing the tip-film distance. This tendency is similar to that of an isolated Ag tip above a glass substrate^34^.

For the tip-film distance of 2 nm, the spatial resolution can be 6.5 nm which is very important for nanoscale molecular imaging. The spectral resolution of TEF can be represented by FWHM. As shown in Fig. 5, the TEF spectral resolution increases rapidly as increasing the tip-film distance. For the tip-film distance more than 2 nm, the resolution can be around 10 nm meaning that the spectral resolution of TEF becomes better with increasing of tip-film distance. The simulated spectral resolution of TEF is agreement with the previous experimental result where a tungsten tip was used above a gold substrate and the H_2_TBPP molecule was chosen as the analysis object^23^. For *in-situ* TERS and TEF measurement, we should choose the suitable tip-film distance for balancing the enhancement factor and the resolution of the both.

In order to verify our idea, we propose an experimental scheme for *in-situ* measurements of TERS and TEF shown in Fig. 1. A confocal spectroscopy system is used to measure the TERS and TEF spectra of R6G molecule *in-situ*. The 532 CW laser and the 633 nm He-Ne laser with 50X objective are used in the experiment, where the 532 nm laser is used to excite molecular fluorescence signals, and the 632.8 nm laser is used to excite the Raman signal. The Raman and fluorescence spectra can be detected using a CCD detector. The experiments will be conducted in the future.

The interaction between vibration modes and the electric field is a critical issue in Raman and fluorescence spectrum. Electromagnetic field enhancement is generally accepted as the dominant contribution to the final enhanced Raman signal. Other action mechanisms also help improve the final Raman signal, such as the most reported photoinduced charge transfer based chemical enhancement. Besides, the electric field gradient effect also contributes to the infrared active vibration modes in Raman spectrum^19^. Hence, the final enhancement of Raman signal is the results of many enhancement mechanisms. Excitation and emission enhancement are considered to contribute to the enhanced fluorescence signal. Like Raman enhancement, the electromagnetic field enhancement, which strongly affects the excitation and emission enhancement, is the dominating factor for the final fluorescence enhancement. In this paper, our purpose is to design optimal instrument to realize the combination of TERS and TEF techniques, but not exploring the underlying physical mechanism that can be found in quite lots of excellent jobs^5^,^28^,^29^. It is of very importance to find the most optimal TERS and TEF configuration to develop multiple functional nano-spectroscopy through which Raman and fluorescence can be precisely measured *in-situ* at the nanoscale level. In the whole work, we built our discussion and analysis from the electric field enhancement point of view, which is regarded as the dominating factor the final enhanced Raman and fluorescence signals.

In this paper, we theoretically proposed a model for *in-situ* measurements of TERS and TEF of dye molecules. The best tip-film distance for *in-situ* measurements of TERS and TEF is 2 nm where the spatial resolution of TERS is 6.5 nm and the spectral resolution of TEF is 10 nm. Our works provide a prospective avenue to *in*-*situ* measure TERS and TEF at the nanoscale level.

## Methods

The 3D-FDTD method was employed to calculate the LSPR-related optical properties of TERS and TEF for the coupled tip-film system (FDTD Solutions 8.7, Lumerical Solutions, Vancouver). The simulation region is set at 1000 nm x1000 nm x1000 nm in 3D. Perfectly matched layers (PML) boundary conditions were used on all boundaries for all simulations. To accurately simulate the nanogap between the tip and film, the mesh size was set to be 0.25 nm. Nonuniform FDTD mesh method was used to save the computation resources and simulation time. Optical constant for silver was taken from ref. 35. The relative dielectric function of R6G adsorbed on Ag film was taken from ref. 25.

Once the molecule is excited, it will behave an oscillating electric dipole and subsequently radiates. Here we used an electric dipole source to model the excited molecule and calculated the nonradiative and radiative decay rate enhancement. Specifically, we have the relationship between the decay rate enhancement (γ/γ_0_) and the radiated power enhancement (P/P_0_)^36^,


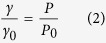


where γ and P is the decay rate and the radiated power in the coupled tip-film system, respectively; γ_0_ and P_0_ is the decay rate and the radiated power in free space, respectively. In the calculations, two monitor boxes were used to calculate the total (P_tot_/P_tot_,_0_) and radiated (P_r_/P_r_,_0_) power enhancement of the electric dipole in the tip-film system, and thus the total decay rate enhancement (γ_tot_/γ_tot_,_0_) and radiative decay rate enhancement (γ_r_/γ_r_,_0_) can be obtained according Eq. 2. The nonradiative decay rate enhancement (γ_nr_/γ_nr_,_0_) can be obtained by subtracting the radiative decay rate enhancement from the total decay rate enhancement. The details of calculation method can be found anywhere^37^,^38^,^39^.

## Additional Information

**How to cite this article**: Meng, L. *et al.* A Nanoplasmonic Strategy for Precision *in-situ* Measurements of Tip-enhanced Raman and Fluorescence Spectroscopy. *Sci. Rep.* **6**, 19558; doi: 10.1038/srep19558 (2016).
